# Big Data in Designing Clinical Trials: Opportunities and Challenges

**DOI:** 10.3389/fonc.2017.00187

**Published:** 2017-08-31

**Authors:** Charles S. Mayo, Martha M. Matuszak, Matthew J. Schipper, Shruti Jolly, James A. Hayman, Randall K. Ten Haken

**Affiliations:** ^1^Department of Radiation Oncology, University of Michigan, Ann Arbor, MI, United States

**Keywords:** big data, trial design, randomized controlled trials, informatics, analytics

## Abstract

Emergence of big data analytics resource systems (BDARSs) as a part of routine practice in Radiation Oncology is on the horizon. Gradually, individual researchers, vendors, and professional societies are leading initiatives to create and demonstrate use of automated systems. What are the implications for design of clinical trials, as these systems emerge? Gold standard, randomized controlled trials (RCTs) have high internal validity for the patients and settings fitting constraints of the trial, but also have limitations including: reproducibility, generalizability to routine practice, infrequent external validation, selection bias, characterization of confounding factors, ethics, and use for rare events. BDARS present opportunities to augment and extend RCTs. Preliminary modeling using single- and muti-institutional BDARS may lead to better design and less cost. Standardizations in data elements, clinical processes, and nomenclatures used to decrease variability and increase veracity needed for automation and multi-institutional data pooling in BDARS also support ability to add clinical validation phases to clinical trial design and increase participation. However, volume and variety in BDARS present other technical, policy, and conceptual challenges including applicable statistical concepts, cloud-based technologies. In this summary, we will examine both the opportunities and the challenges for use of big data in design of clinical trials.

## Introduction

A primary objective of clinical research is gaining knowledge from studying a subset of patients which can then be applied to a much wider group of patients to improve care. In routine practice, patient care is delivered within a rich background of intrinsic and endemic confounding factors and biases associated with practices and patients. Clinical research methodologies are challenged to accurately delineate specific relationships and be relevant to routine practice.

Optimal trial design methodologies have a long history of debate within the medical field (1–15). Recently, there has been substantial growth in the number of academic groups investing in development of big data analytics resource systems (BDARSs) to support practice quality improvement (PQI) and translational research (TR) applications in radiation oncology (16, 17). BDARSs aggregate clinical data from multiple systems including electronic health records (EHRs), Radiation Oncology information systems (ROISs), treatment planning systems (TPSs), and others into common location designed to support analyzing this data to improve patient care. Our objective in this presentation is to explore how these big data efforts might intersect with trial design methodologies to augment or extend these approaches.

## Randomized Clinical Trials

Randomized controlled trials (RCTs) provide the highest ranked level of evidence for delineation of causal relationships between treatment results and outcomes. Using a design methodology that meticulously minimizes and controls variation encountered in routine practice, RCTs are designed for statistical rigor. They have high internal validity for selected constraints and treatment delivery conditions specified in the trial design. RCTs are well incorporated into clinical and research systems. Systems for funding, management, and infrastructure supporting collaborative trials research are oriented to RCTs. However, RCT’s also have challenges including: reproducibility, generalizability, cost, external validation, and delay (1, 2, 14). Meta-analysis of individual patient data addresses some of these challenges of any single trial. In particular, results of a meta-analysis of multiple clinical trials will generally be more reproducible, generalizable, and have greater external validity. However, they also have greater delay and cost than any single trial. Additionally, they are still based on the population of patients who actually enroll in clinical trials which may not be fully representative of a broader patient population.

### Reproducibility

Multiple, independent measurements demonstrating reproducibility of results are strong evidence for the validity of the result. Difficulty in reproducing results for RCTs is a concern in the community and for the National Institutes of Health (3). Observational studies are ranked lower than RCTs in level of evidence, but frequently utilize larger number of patients. Some researchers have demonstrated greater consistency among observational studies than findings consistent with RCTs (2, 4, 5). In an analysis comparing results of independent RCTs (45) to independent, well-designed observational studies (44) spanning five clinical research topics, Concato demonstrated more inconsistency in RCT, and much tighter confidence intervals for the observational studies which included larger number of subjects (2). In an early meta-analysis Horwitz examined 200 RCTs spanning 36 topics in cardiology and gastroenterology highlighting conflicting results. He found that complex design and inconsistencies in clinical execution and therapeutic evaluation undermined reproducibility (4). In radiation oncology, complex single institution trials may require significant redesign to reduce complexity, such as in the case of translating the University of Michigan’s PET adaptive lung cancer trial to a cooperative group trial run through RTOG (18, 19). Additionally, compared with pharmacologic interventions, technique-based interventions in Radiation Oncology as in Surgery, introduce added complexities sensitive to skill of individual practitioners, and evolution of technique over the period of the trial as experience is acquired.

### Cost

Effort required for collection and aggregation of data frequently falls outside the range of routine clinical practice. Interfaces to EHRs, ROISs, and TPSs typically require manual inspection of all to synthesize, extract, and report required trial data.

### Generalizability

Complexity and cost of implementing trials work against recruitment of large numbers of patients and introduces selection bias for patient cohorts with geographic, insurance, and medical history profiles commensurate with treatment at medical centers that also have sufficient resources to participate in trials. This selection bias can become dangerous when the RCT result is applied to an underrepresented group of patients that were not well represented in trial enrollment and whose disease may not respond to the experimental treatment. In addition, RCTs are typically designed to test a drug or specific intervention in a patient cohort with strict eligibility criteria. In many cases, RCTs are testing these interventions in a small subset of patients in larger disease sites. So, even after a positive trial, the number of patients that the results of an RCT may apply to, could be relatively small. However, this does not prevent the community from applying the intervention to a larger cohort of patients, making future observation studies potentially washed out or negative due to inappropriate use of the trial results.

As more data on genomic variations across patients and tumors becomes available, it is also possible that the results of certain positive trials could be driven by strong positive result in a previously unknown subset of the population. Without further study and patient classification by BDR, the ability to further analyze these trials is lost.

### Infrequent External Validation

If an objective of funding RCTs is to improve care for a broader segment of the population, then demonstrations of external validation are needed. Due to a variety of factors, RCTs suffer from low rates of external validation. Larger RCT series with multiple studies testing similar regimes, such as accelerated whole breast irradiation (6, 7) are the exceptional case where RCTs can lead to sweeping practice changes and updated national guidelines. However, smaller RCTs, especially those run in a single institution setting, are rarely validated in an external cohort due to complex design, cost, and loss of equipoise after the initial trial is published.

One reason for this may be that testing a trial concept for extensibility to and validity in the “real world” of routine clinical practice is rarely a priority in trial design. Therefore, RCTs continue to include a much, much smaller number of patients and less variable clinical practices than represented by the majority of patients treated.

As more and more biomarker and image driven treatment selection is incorporated into trials, this lack of external validation will only become worse. Not only will the validation studies not be possible due to the lack of knowledge and resources to run the trial, but specific nuances of image analysis and bio-specimen testing/handling, may be unavailable or irreproducible. National clinical trial resources and core facilities will assist in this area for larger cooperative group studies, but this remains an issue for single institution studies.

### Delay

Clinical trial infrastructure, both at individual institutions and cooperative groups, is organized in such a way that trials go through a number of steps to ensure that trials are of sufficient potential benefit to the patient or population, are able to be funded appropriately, and are designed properly. While these steps are essential, it also means that the initiation of a trial is delayed by even years before starting.

Almost one-fifth clinical trials even at large centers are “slow-accruing” (14). Thus, once a trial opens, the study question may no longer be as relevant as it was when the concept was first initiated. Expense of tests and staff to carry out the RCT may limit resources needed for accrual into the trial. Use of manual rather than standardized electronic means at point of care—point of data entry impede aggregation from multiple institutions. Managing logistics of clinical process flows and mechanisms for data aggregation for RCTs that differ from those used for the majority of off-protocol patients add to cost and slow accrual.

## Synergies in Constructing Big Data Systems and Supporting Clinical Trials

Rather than replacing RCTs, we posit that BDARSs will present resources and methodologies that can be incorporated into design of RCTs to augment and extend them to address the issues outlined above. Assuring that data elements needed for BDARSs are routinely aggregated using methodologies that assure accurate electronic extraction is also synergistic with objectives for clinical trials and observational studies. Construction of effective BDARSs includes development and use of standardizations that can be practically fitted into clinical practice. Coordination with multi-disciplinary groups to clean point of care—point of data entry processes to support BDARSs is extensible to these groups for entry of data elements necessary for clinical trials. Standardizations in designation of key data elements, nomenclatures supporting exchange, and clinical processes improving accurate are vital to these efforts.

### EHR Templates

For example, our BDARS, the University of Michigan Radiation Oncology Analytics Resource (M-ROAR), requires accurate data on provider reported toxicities, recurrence, performance status, etc. (18). Examining the work flows of care providers, the most consistent point of entry is provider notes in the electronic health record (EHR). Our EHR, EPIC, does not provide quantified fields for these key data elements. However, with development of M-ROAR to enable use of the full text of encounter notes, options for standardizing text entry to enable accurate, automated electronic extraction became viable solutions.

The EHR does provide means create templates that regularize text entry of information. In that EHR system, these are known as Smart List and Smart Phrase objects. Smart List objects allow defining a tab activated drop down list of serializable options to be inserted in the text field of a clinical note. Smart phrases are used to assemble sets of smart lists embedded with other standardized text.

We developed a standardized schema for representation of key data elements in text fields utilizing these smart objects to regularize data entry across providers. With this schema standardization, software tools known as regular expressions can be used to accurately extract key data elements from the text of clinical encounter notes. This is carried out in high volume for all patients.

The schema developed demarking key data elements are illustrated below. Highlighted text indicates characters with specific interpretations. Italicized text indicates place holders for specific information types.

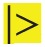
*Key Data Element* 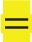 *Value* 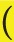
*qualifying information*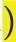 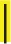 *supplemental element* 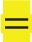 *value*
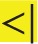

**Figure 1:**
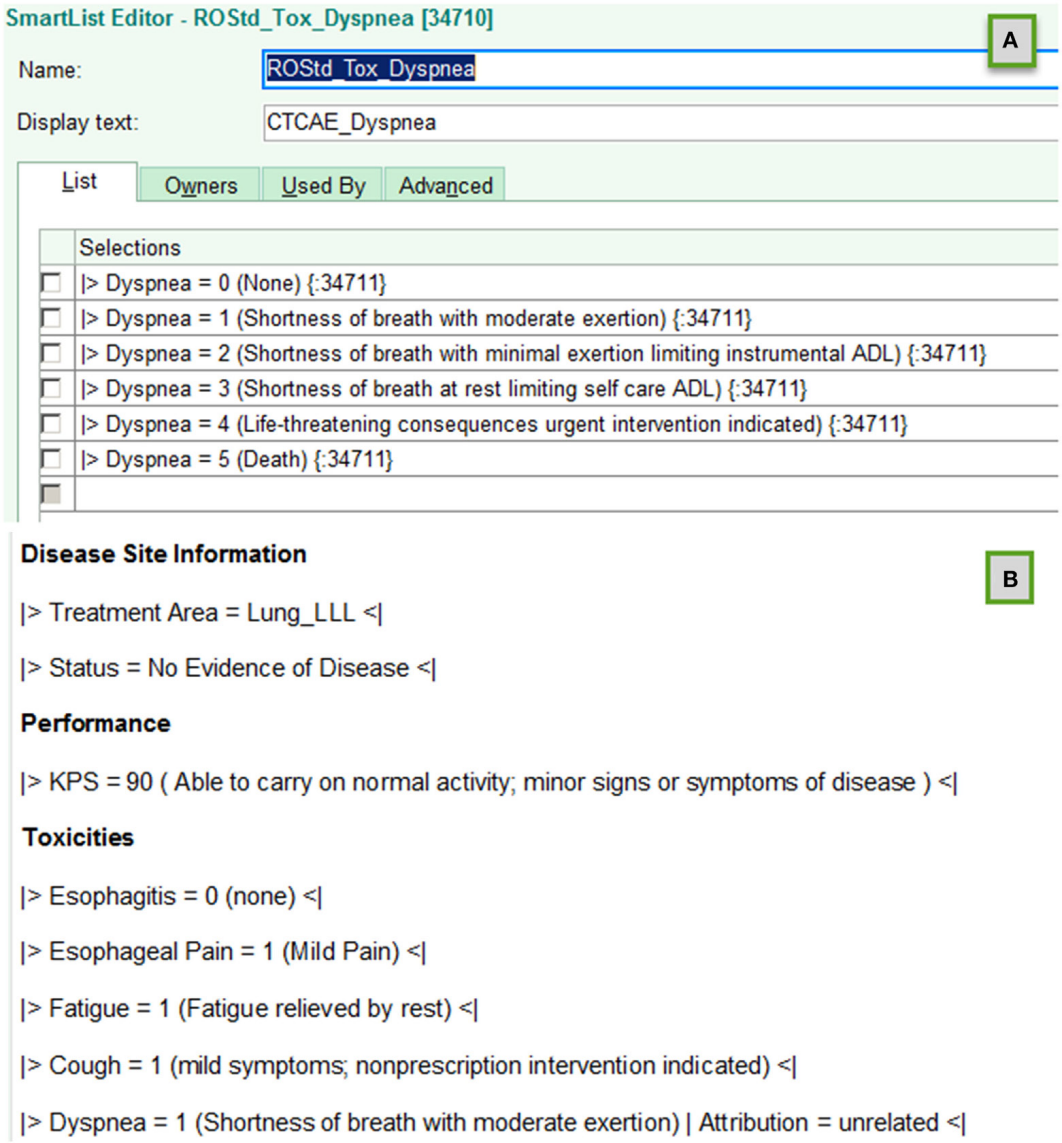
Examples of using template objects in electronic health records to implement data entry standardizations that support accurate automated electronic extraction are shown for **(A)** smart list and **(B)** smart phrase objects in an EPIC environment.

Figure 1 illustrates creation of smart list objects using this schema. The 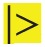 and 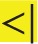 character combinations delineate the beginning and the end of a key data element. The text to the left of the 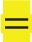 sign following 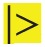 is a standardized name for the key data element; the text to the right indicates the value assigned to the data element. Parenthesis characters, 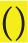, are used to delineate optional commentary information. The bar symbols, 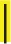, demark entry of optional supplemental item/value pairs related to the key data element. Four examples of schema valid text fields are listed below.

|>Xerostomia = 1<|
|>Dysphagia = 2 (Symptomatic, altered eating swallowing) | Attribution = related to treatment<|
|>Recurrence = Local<|
|>Performance:KPS = 90<|

The standardized schema assures accurate identification of key data elements and component information elements. Together with definition of a standardized data dictionary of key elements, supplemental information items and allowed values, the standardized schema provides a flexible but fully defined means to accurately and electronically extract information needed for BDARSs.

When a clinical trial is implemented, additional key data elements may be needed. If the EHR is the optimal point of care-point of data entry mechanism, then the data dictionary is extended, and new smart list/smart phrase objects are constructed using the standardized schema developed to support extractions for the BDARS.

Note that while access to TPS and ROIS data is routine in most Radiation Oncology clinics, access to EHR data varies widely among institutions. Considerable cooperation between the EHR vendor and the institutional IT groups controlling access with end users is required. Introduction of standardizations, like that defined above, increases the value of the enterprise data stores for both vendors and IT groups as well as for end users. However, these standardizations only arise and become incorporated into routine practice if end users are enabled to access and use the data. This is especially important for community clinics, where the majority of patients are treated.

### Optimized Clinical Process Flow Using Existing Systems

For several key data element categories, ROISs or TPSs may be optimal point of care-point of data entry systems. Optimizing clinical process to assure availability of these elements for all patients supporting the BDARSs also eliminates extra efforts to acquire these elements when needed for clinical trials.

For example, by modifying clinical process flows to implement a standardized approach for entry of diagnosis and staging information along with explicit linkages to treatment course, both the BDARS and clinical trials are supported. In another example supporting the BDARS, we modified our clinical process to assure routine creation of as treated plan sums to enable automated extraction of course cumulative dose volume histogram (DVH) curves reflecting cumulative doses for the plans and actual number fractions treated. In addition, the standardized nomenclature recommendations of AAPM TG-263 for targets and normal structures were adopted to assure correct identification of structures in extract, transform, and loads (ETLs) of DVH curves.

Patient reported outcomes aggregation required modification of clinical process flows and staffing as well as collection technology. With subsequent completion of the informatics circle to ETL PRO data into M-ROAR, the PRO data became available for large volume analysis. With that step, the mechanisms used for gathering PROs for M-ROAR, could plausibly be extended to support gathering analogous information for patients on RCTs.

### Multiple Institutions

Ability to aggregate key data elements, including survival, recurrence, and toxicity, is challenged when patients do not return for follow-up or shift away from the academic center delivering specialized care back to their local community hospital for ER and continuing care visits. Fully understanding therapeutic outcomes requires longitudinal follow-up data over many years. Scalable, automated solutions are technically feasible, but requisite contractual relationships and PHI protection compliance mechanisms are not. Health care policy efforts to improve continuity of care will in the long run benefit both BDRs and RCTs.

The regulatory and institutional compliance office constraints arising from the Health Insurance Portability and Accountability Act (HIPAA) are important for protecting sensitive, personal information of patients from misuse. However, HIPAA can be a double edged sword. Ability to utilize information gained from prior patients from multiple institutions to improve treatments of future patients is a desirable use. Current views of how to implement the intent of HIPAA often prevent reaching this potential. Finding a middle ground that affords needed protections, while also enabling the benefits of multi-institutional datasets is a vital area of collaboration between patient advocacy groups, legislators, regulatory groups, and researchers.

## Using Big Data to Augment Trial Design

As BDARSs emerge, are integrated with EHRs, ROISs, and TPSs and applied to all patients treated, they present resources for improving trial design. Successfully carrying out this integration requires navigating multi-disciplinary, multi-stakeholder clinical processes needed to achieve access, and implement standardizations (20, 21). Building standardizations and automations into systems reduces the amount of manual effort required to enter and extract data, lowering cost. In addition, wider adoption of standardizations and templates and applications supporting BDARSs lowers resource thresholds for participation in RCTs. This should translate to increasing participation in RCTs.

By proactively identifying and incorporating BDARS supporting standardizations, researchers designing trials can improve curation and reproducibility. Standardizations reduce complexity introduced by variability and increase reliability of consistency checks on inputs and outputs. Use of these standards in routine clinical care and in RCTs makes possible development of sharable automated curation algorithms to flag outliers or longitudinal variation in data entry that may signal errors.

For example, AAPM’s Task group 263 on Standardization of Nomenclature for Radiation Therapy defined standards for naming of target and normal structures as well as defining a schema for representing DVH metrics. The task group of 57 members representing, a broad range of roles (e.g., physician, physicist, vendor), professional societies (e.g., AAPM, ASTRO, ESTRO), clinic types (e.g., academic, community practice), and specialty groups (e.g., IHE-RO, DICOM, NRG) to meet common needs of RCTs and routine practice (22). This standard has been adopted by NRG in designing new trials (23). By adopting this standardization into routine practice, effort to prepare data for RCT trial aggregation sites or use in local PQI and TR is reduced.

By designing trials to utilize BDARSs as the optimal aggregation system rather than manual one-by-one extraction from EHRs, ROISs, and TPSs, ability to extend trial results to routine practice and later to carry out validation studies is improved. With this approach, by utilizing BDARS aggregations up front when there are resources for introducing the RCT, then the infrastructure for follow-on efforts is largely in place. In addition, by identifying and fixing “pinch points” in clinical processes to support the BDARS, highlighting practice sensitive data elements affecting RCTs and ability to design trials with intent to incorporate external validation is improved.

Further, with automated aggregation of multiple data elements the range of confounding factors that can be tested in the trial increases. In addition, standardization and automation extended across multiple centers increases ability aggregate enough patients to examine rare events.

## Considerations in Observational Studies

One of the main challenges to learning from BDARSs is the potential for confounding. In RCTs, the randomization ensures that patients receiving each of the randomized treatments will, on average, be similar with respect to any baseline variable. In observational datasets, there often exist selection biases such that patients receiving two different treatments have different distributions of a variable that may be related to an outcome of interest.

There are a number of statistical approaches to assessing and accounting for confounders. A simple approach is to use multivariable regression models in which potential confounders are included as covariates in addition to treatment. A generally preferable approach is to use propensity scores as weights (inverse probability of treatment), strata, or matching variables (24). Using propensity scores as weights creates a “synthetic” population of outcomes in which both treatment groups have similar distributions of any measured confounders. In this sense, it mirrors an RCT. Both multivariable regression models and propensity methods account only for measured confounders. In some settings, there may be unmeasured confounders.

Instrumental variable analysis (IVA) (25) represents an approach which can provide valid treatment effect estimates in the presence of unmeasured confounding if certain assumptions are met. IVA analyses rely on the selection of an “instrumental” variable that is correlated with treatment and meets other conditions. Importantly, these conditions cannot be verified empirically from the data so that selection of an instrument must be based on subject-matter knowledge.

## Using Big Data to Extend Trial Design

**Figure 2:**
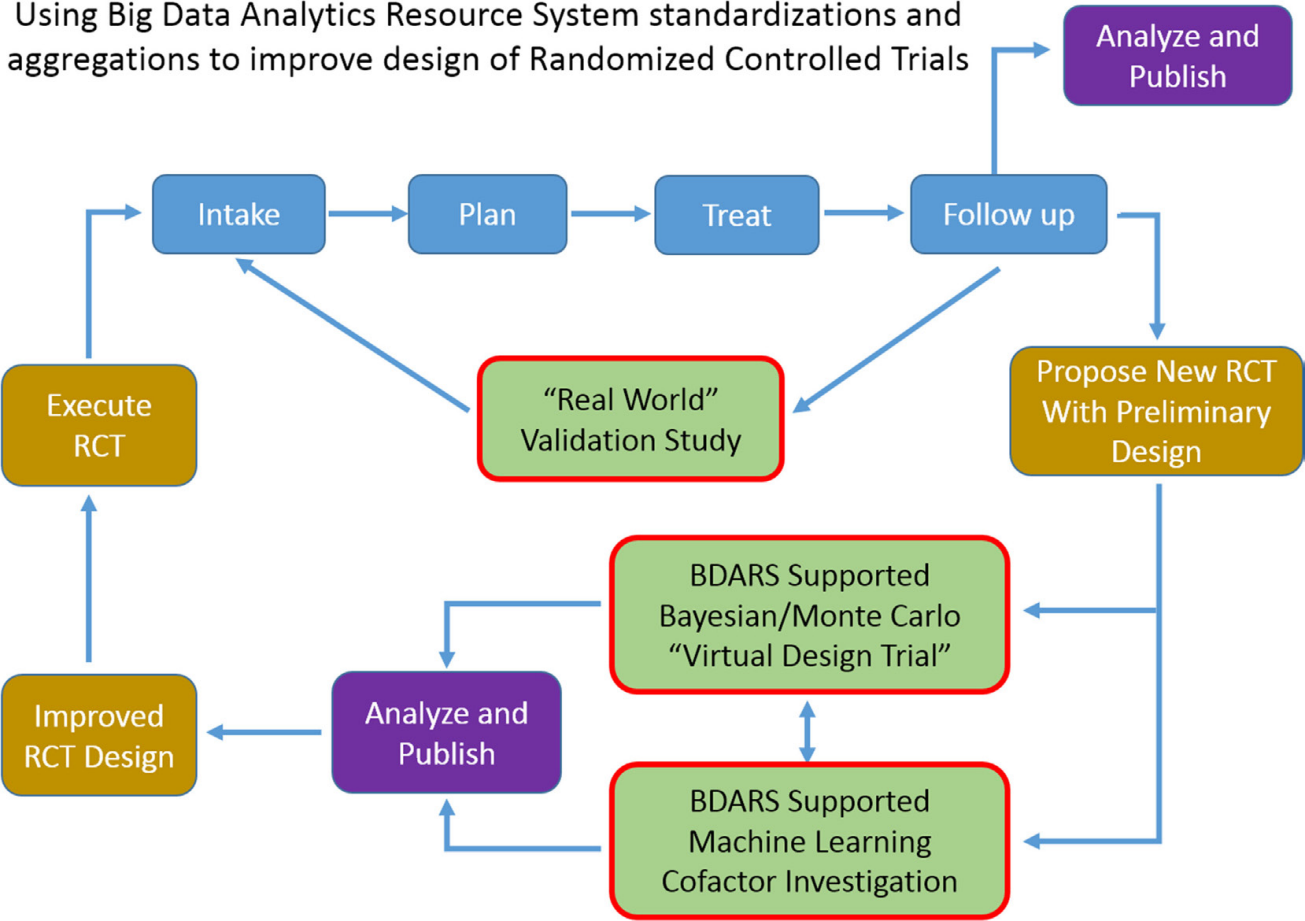
As use of big data analytics resource system expands, ability to carry out multi-institutional validation studies, and to improve randomized controlled trial design with “virtual design trials” will expand.

Increase in availability of BDARS also presents several opportunities to extending clinical trial design methodologies or to generate RCT hypothesis fueled by large, preliminary observational studies. BDARS make distributions for a wide range of treatment and diagnostic parameters readily available. These distributions can be utilized to carry out “virtual design trials” ahead of designing the RCT (Figure 2).

For example, in designing a trial aimed at investigating the co-dependence of a chemotherapy regime used in conjunction with an SBRT dose escalation strategy for lung cancer patients, historic data could be used to examine distributions, and cross-correlations of demographic, radiation, and chemo therapy treatment parameters, dosimetric, and laboratory values, survival, recurrence, provider reported toxicities, and patient reported outcomes. With the distributions and inter-relationships characterized, variations as anticipated from the proposed trial can be simulated with Monte Carlo and Bayesian methods to better anticipate confounding interactions and to optimize design decisions. Machine learning approaches can be used to leverage the wide range of data element categories contained in BDARS to identify unanticipated interactions and dependencies that should be considered in the RCT design. When the BDARS contains data on charges and procedure codes, ability to improve projecting budgets for the trial is improved. This approach puts examination of the confidence intervals of key parameters and implications for the study up front using actual data rather using hypothetical projections and having to adjust the RCT after it is started.

Prior to conducting an RCT, investigators could utilize BDARSs to more precisely understand characteristics of patients with a particular type of cancer or of patients being treated with a certain treatment. This knowledge could then be translated into the design of the RCT to ensure that the patients enrolled on the RCT are reflective of the intended population. This could mean, for example, that enrollment would be stratified by subgroups. A key step in designing RCTs is selection of sample size. Key drivers of sample size include effect size estimates as well as estimates of variance. There is much room for improvement in how these parameters are selected in the design stage and BDARSs could be utilized to estimate them more precisely and accurately. BDARSs could also be used to accurately estimate the number of eligible patients and hence likelihood of completing accrual within a timely fashion.

After an RCT is completed, BDARSs could be utilized to assess uptake of the “winning” treatment and importantly whether the results in actual clinical practice are similar to those observed in the RCT. One reason for discrepancy has to do with how the treatments are implemented. Treatments such as IMRT are complex and can vary substantially in important details such as normal tissue constraints. If these variables are captured as part of the BDAR, then the source of discrepant results can be sought in discrepant implementations.

In addition, ability for a site proposing an RCT to carry out this analysis demonstrating the potential of the proposed RCT, either as a single- or multi-institutional effort, provides a low cost means of testing the potential value of the RCT and focuses funding on efforts with significant likelihood of success. Publication of these virtual trial results ahead of implementing the actual RCT would place specific and focused discussion of the trial design and potential weaknesses ahead of implementation.

## Conclusion

The recent surge in big data initiatives in health care is expected to have a positive impact on clinical trials. Increased standardization of common data elements and nomenclature should assist in streamlined trial design and exchange of data. Standardize between trials and will allow easier multi-study analysis. Standardization and quality improvement efforts go hand in hand with a maturing big data infrastructure providing collateral benefits to data curation for RCTs (24, 26).

The quality and power of observational studies will increase tremendously as use of BDARS increases. Addition of standard outcomes measurements and patient reported outcomes to clinical databases will widen the range for which observational studies are deemed high quality evidence. While BDARS-based observational studies will not eliminate need for RCTs, they can be anticipated to raise expectations for level of evidence thresholds required from RCTs and prompt more frequent validation studies.

Granting agencies may note dividends from BDARS supporting standardizations and ETLs for lowering cost and improving RCT design. Funding for virtual design trials using Bayesian and Machine Learning methodologies will promote standardizations and growth of BDARS that will ultimately support and improve the quality of RCTs.

## Author Contributions

The authors have participated in discussion of concepts presented in the manuscript, writing, and/or review of the manuscript.

## Conflict of Interest Statement

The authors declare that the research was conducted in the absence of any commercial or financial relationships that could be construed as a potential conflict of interest.
